# Supplementary material for: MIRO1 Is Required for Dynamic Increases in Mitochondria-ER Contact Sites and Mitochondrial ATP During the Cell Cycle
Source: Cells. 2025 Mar 22;14(7):482. doi: 10.3390/cells14070482 (PMC11988184; doi:10.3390/cells14070482)
Supplement: Supplementary file 1 [file cells-14-00482-s001.zip › cells-3394683-supplementary.pdf]

cells MDPI

Article

MIRO1 is required for dynamic increases in mitochondria ER contact sites and mitochondrial ATP during the cell cycle

Benney T. Endoni[1,2], Olha M. Koval[1], Chantal Allamargot[3], Tara Kortlever[1], Lan Qian[1], Riley J. Sadoski[1], Denise Juhr[1], Isabella M. Grumbach[1,4,*]

Supplementary Materials: The following supporting information can be downloaded at: www.mdpi.com/xxx/s1, Supplementary Figures S1-S9.

Citation: To be added by editorial staff during production.

Academic Editor: Firstname Lastname

Received: date
Revised: date
Accepted: date
Published: date

Copyright: © 2024 by the authors. Submitted for possible open access publication under the terms and conditions of the Creative Commons Attribution (CC BY) license (https://creativecommons.org/licenses/by/4.0/).

	Vendor	Catalog Number	Species	Application
Antibodies				
MIRO1	Atlas	HPA010687	Rabbit	WB, PLA
IP3R	Cell Signaling technology	8568S	Rabbit	WB, PLA
GRP75	Cell Signaling Technology	3593S	Rabbit	WB
GRP75	Santa Cruz	sc-133137	Mouse	PLA
VDAC1	Cell Signaling Technology	4661S	Rabbit	WB
VDAC1	Abcam	Ab186321	Mouse	PLA
FACL4	Abcam	ab155282	Rabbit	WB
VAPB	Proteintech	14477-1-AP	Rabbit	WB
VAPB	Invitrogen	MA5-24348	Mouse	PLA
PTPIP51	Proteintech	20641-1-AP	Rabbit	PLA
GAPDH	Cell Signaling Technology	2118S	Rabbit	WB
Cyclin D	Cell Signaling Technology	2922S	Rabbit	WB
Cyclin E	Cell Signaling Technology	20808S	Rabbit	WB
PDH	Cell signaling Technology	3205S	Rabbit	WB
p-PDH	Cell Signaling Technology	31866S	Rabbit	WB
MCU	Cell Signaling Technology	14997S	Rabbit	WB
MICU1	Cell signaling technology	12524S	Rabbit	WB
BiP	BD Transduction Laboratories™	610978	Rabbit	WB
XBP1	Abcam	ab37152-1001	Rabbit	WB
CHOP	Cell Signaling Technology	2895T	Rabbit	WB
c-Myc	Santa Cruz	sc-40	Mouse	WB
COX IV	Cell Signaling Technology	4850T	Rabbit	WB
GFP	Thermo Fisher	A11122	Rabbit	WB
Tom20	Cell Signaling Technology	42406	Rabbit	WB

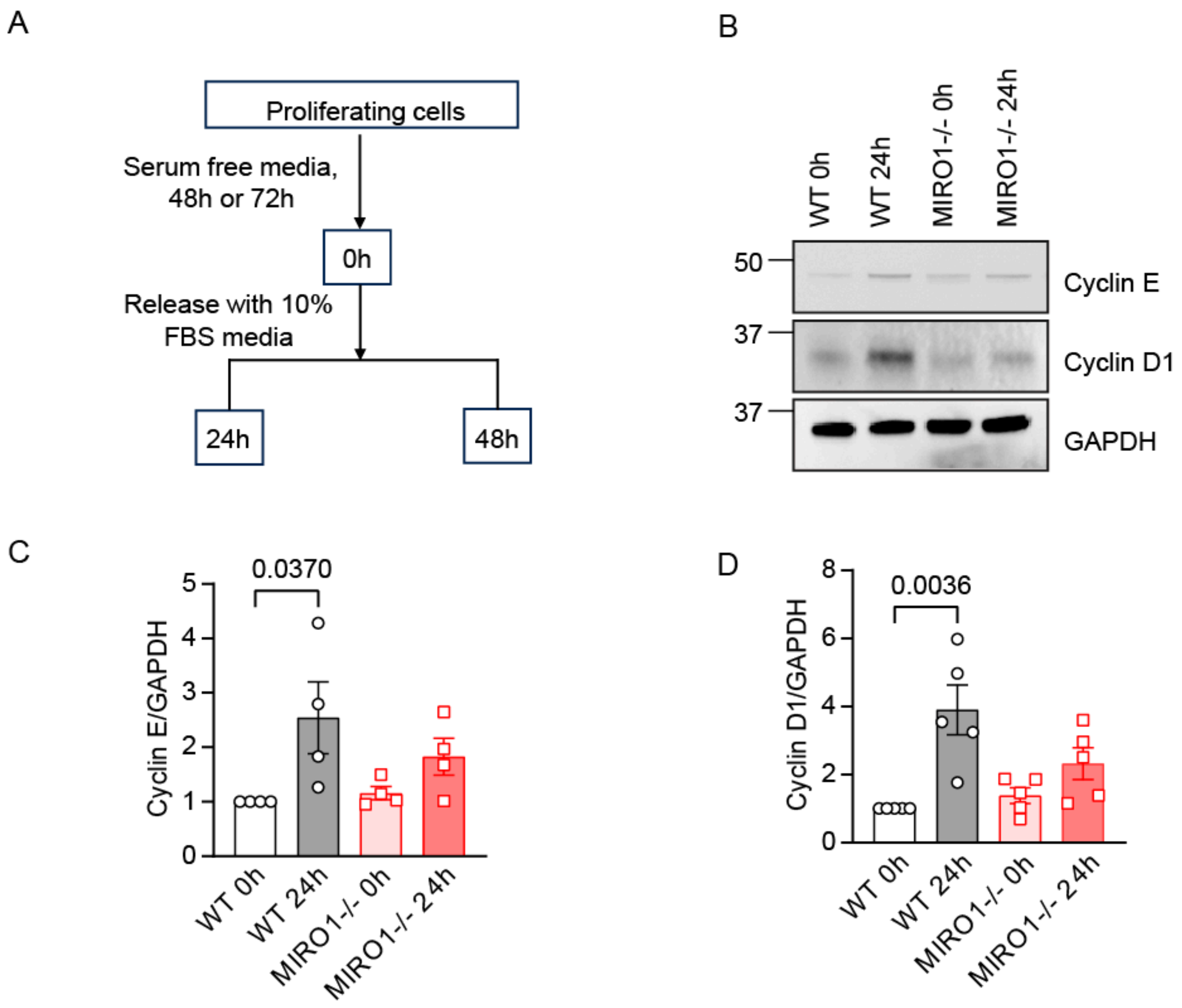

Figure S1: Synchronization of cells and cyclin expression during cell cycle progression.

(A) Schematic of cells synchronization at different stages of the cell cycle. VSMCs or skin fibroblasts were serum starved (serum free media) for 48 or 72 h respectively (0 h), and released into the cell cycle in media supplemented with 10% FBS for 24 h and 48 h.

(B) Representative immunoblots for cyclins D1 and E in WT and MIRO1$^{-/-}$ skin fibroblasts after synchronization in serum-free medium at 0 h, and at 24 h after release from growth arrest in medium containing 10% FBS.

(C – D) Quantification of immunoblots shown in (B). (C) Cyclin E, (D) cyclin D1. The quantification is adjusted to GAPDH; n = 4 - 5 independent experiments.

Data are shown as the mean ± SEM. Analyzed using the Friedman test.

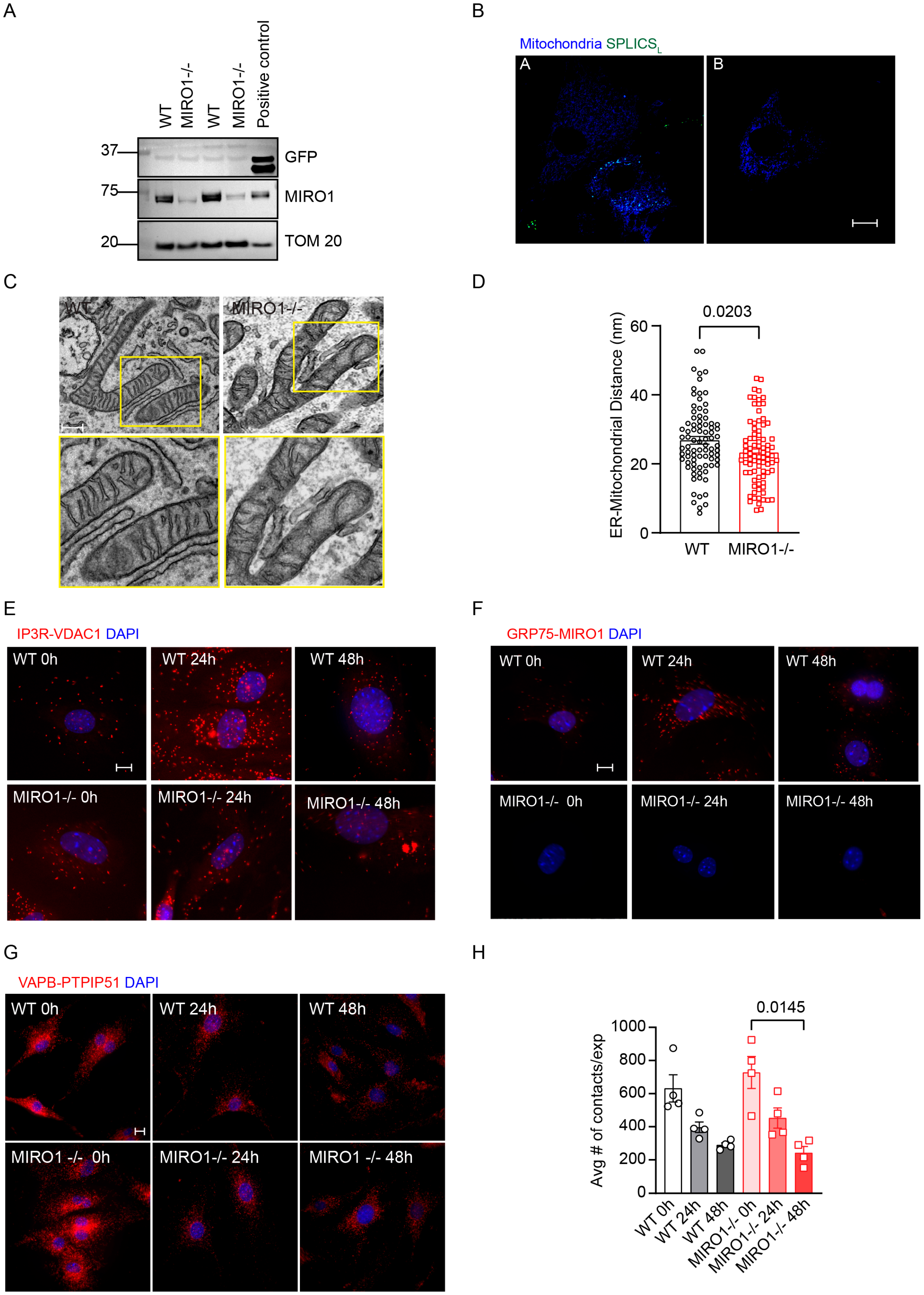

Figure S2: Effect of MIRO1 deletion on SPLICS expression, mitochondria-ER distance, and VAPB-PTPIP51 interaction during the cell cycle.

(A) Immunoblot for GFP in mitochondrial fractions of WT and MIRO1$^{-/-}$ fibroblasts transduced with SPLICS$_L$ for 24 h and HEK cells transduced with mito-GFP (positive control) for 72 h; TOM20 was used as loading control.
(B) Representative confocal images of skin fibroblasts expressing SPLICS$_L$ (A) and nontransduced cells (B). Scale bar = 10 µm
(C) Transmission electron microscopy images of WT and MIRO1$^{-/-}$ VSMCs. Yellow squares depict magnified views of mitochondria and ER. Scale bar = 0.5 µm
(D) Quantification of mitochondrial ER distance. Distance between the two organelles was measured three times and averaged. n = 83 averaged measurements from TEM images of 41 WT and 38 MIRO1$^{-/-}$ cells.
(E) Insets of the representative images of in situ proximity ligation assay (PLA) between IP3R and VDAC1 in Figure 2E. Scale bar = 10 µm
(F) Insets of the representative images of in situ proximity ligation assay (PLA) between GRP75 and MIRO1 Figure 2G. Scale bar = 10 µm
(G) Representative images of in situ proximity ligation assay (PLA) between VAPB and PTPIP51. PLA products are shown in red, nucleus in blue (DAPI). Scale bar = 20 µm, ×40
(H) Quantification of images in (C); n = 4 independent experiments.

Data are shown as the mean ± SEM. Analyses were performed using the unpaired t-test (D) and Kruskal-Wallis (H) test.

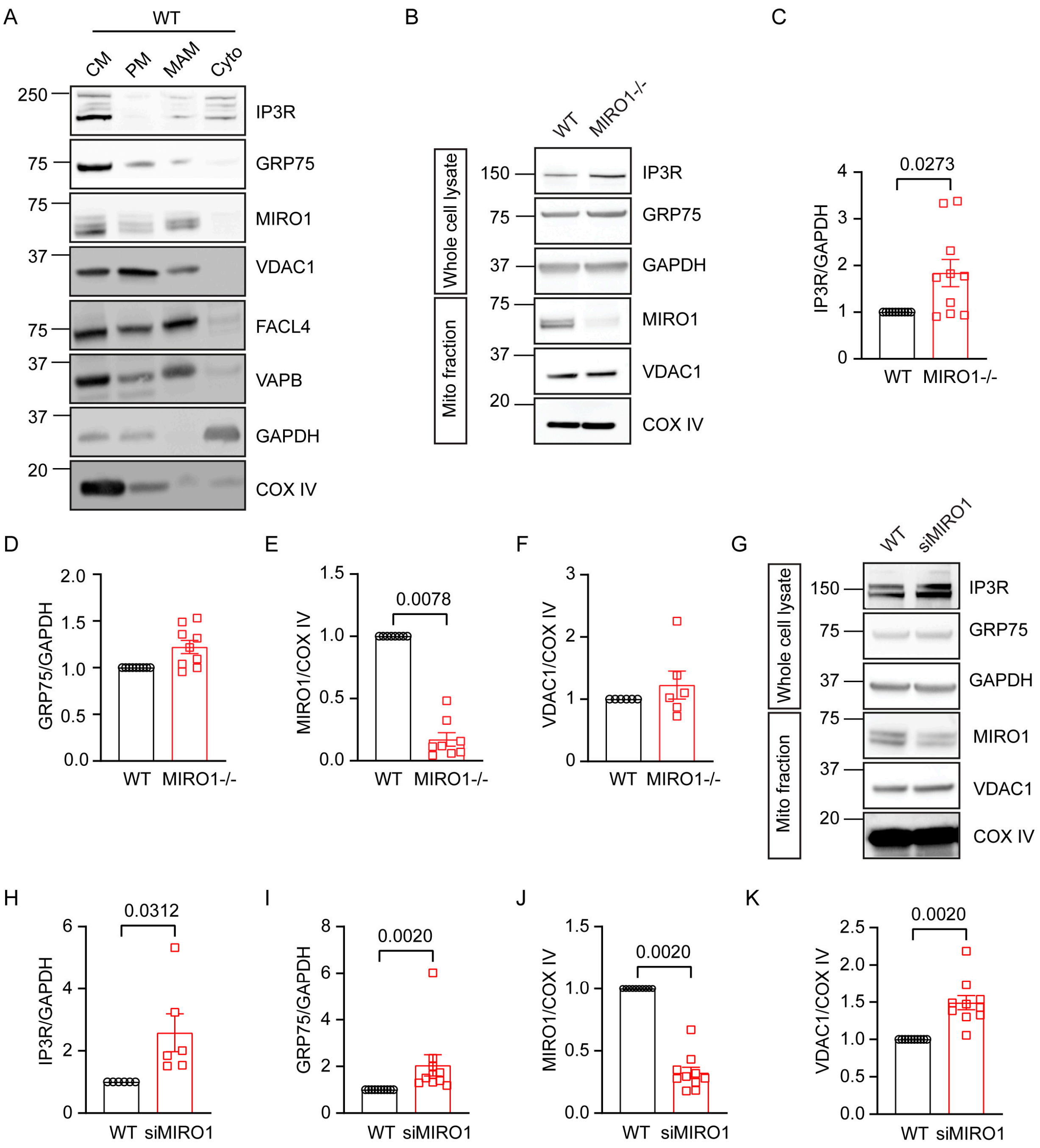

Figure S3: MERCS proteins expression in MAM fractions isolated from WT fibroblasts and lysates from VSMCs and HEK cells.

(A) Representative immunoblots for MERCS proteins in fractions of crude mitochondria (CM), purified mitochondria (PM), mitochondria associated membrane (MAM), and cytosol (Cyto) isolated from WT fibroblasts. Markers for MAMs and ER (FACL4) and mitochondria (cytochrome c oxidase [COX IV]) were examined. COX IV and GAPDH were used as loading controls.

(B) Representative immunoblots of MERCS proteins in whole cell lysates or mitochondrial fractions from WT and MIRO1$^{-/-}$ VSMCs. GAPDH and COX IV respectively was used as loading controls.

(C – F) Quantification of immunoblots shown in (B). (C) IP3R, (D) GRP75 adjusted to GAPDH. (E) MIRO1, (F) VDAC1 adjusted to COXIV; n = 6 – 10 independent experiments.

(G) Representative immunoblots of MERCS proteins expression in whole cell lysates or mitochondrial fractions from WT and siMIRO1 HEK cells. GAPDH and COX IV respectively were used as loading controls.

(H – K) Quantification of immunoblots shown in (G). (H) IP3R, (I) GRP75 adjusted to GAPDH. (J) MIRO1, (K) VDAC1 adjusted to COXIV; n = 6 – 10 independent experiments.
Data are shown as the mean ± SEM. Analyzed using the Wilcoxon test.

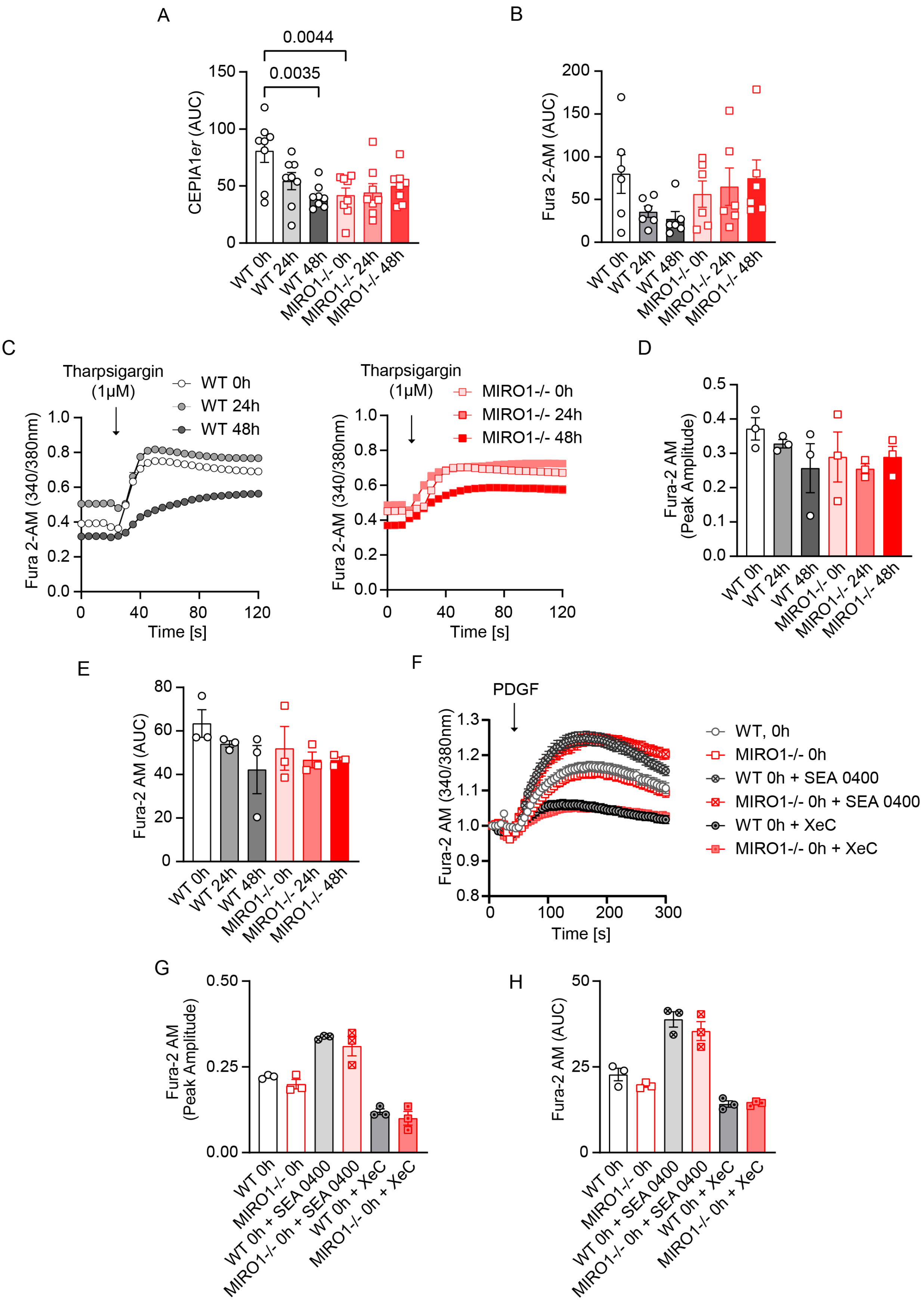

Figure S4: MIRO1 regulates changes in subcellular Ca^{2+} distribution during cell cycle but not NCX-dependent Ca^{2+} extrusion or IP3R function.

(A) Quantification of area under the curve (AUC) for ER Ca^{2+} transients shown in Figure 4A; n = 8 independent experiments.
(B) Quantification of area under the curve (AUC) for cytosolic Ca^{2+} transients shown in Figure 4C; n = 6 independent experiments.
(C) Tharpsigargin-induced cytosolic Ca^{2+} transients assessed with Fura 2-AM in synchronized/growth arrested WT and MIRO1$^{-/-}$ VSMCs at 0 h, and after release from arrest with 10% FBS for 24 h and 48 h. Arrows indicate addition of tharpsigargin (1 µM).
(D) Quantification of peak amplitude of Fura 2-AM recordings shown in (C). n = 3 independent experiments.
(E) Quantification of area under the curve (AUC) shown in (C). n = 3 independent experiments.
(F) Cytosolic Ca^{2+} extrusion was assessed with Fura 2-AM in synchronized/growth arrested WT and MIRO1$^{-/-}$ VSMCs at 0 h following treatment with NCX inhibitor (SEA 0400) or IP3R inhibitor xestospongin C (XeC). Arrow indicates addition of PDGF.
(G) Quantification of peak amplitude of Fura 2-AM recordings shown in (F). n = 3 independent experiments.
(H) Quantification of the area under the curve (AUC) shown in (F). n = 3 independent experiments.

Data are shown as the mean ± SEM. Analyses were performed using the one-way ANOVA (A), Kruskal-Wallis (B, D – E, G - H) tests.

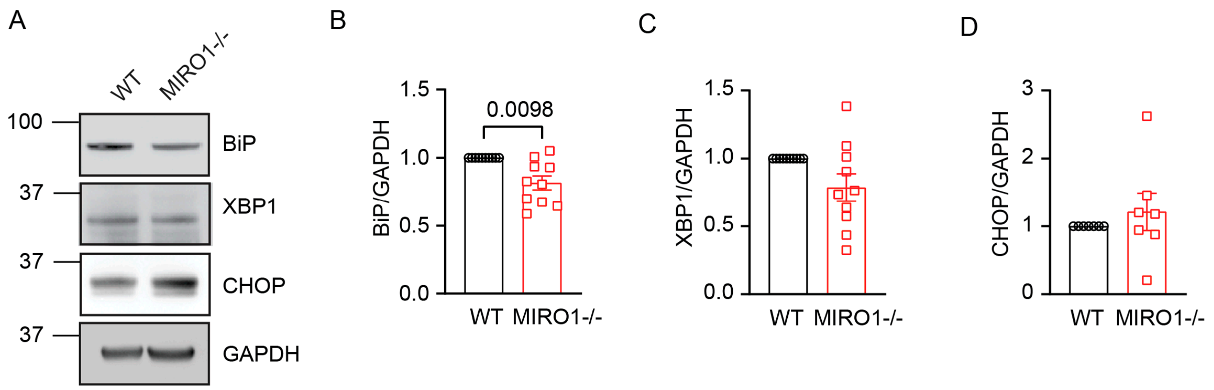

Figure S5: MIRO1 deletion does not affect XBP1 and CHOP levels in VSMCs.

(A) Representative immunoblots for ER stress proteins in whole cell lysates from WT and MIRO1$^{-/-}$ VSMCs; GAPDH was used as loading control.

(B – D) Quantification of immunoblot experiments shown in (A). (B) BiP, (C) XBP1, (D) CHOP, adjusted to GAPDH. n = 7 – 10 independent experiments.

Data are shown as the mean ± SEM. Analyzed using the Wilcoxon test (B - D).

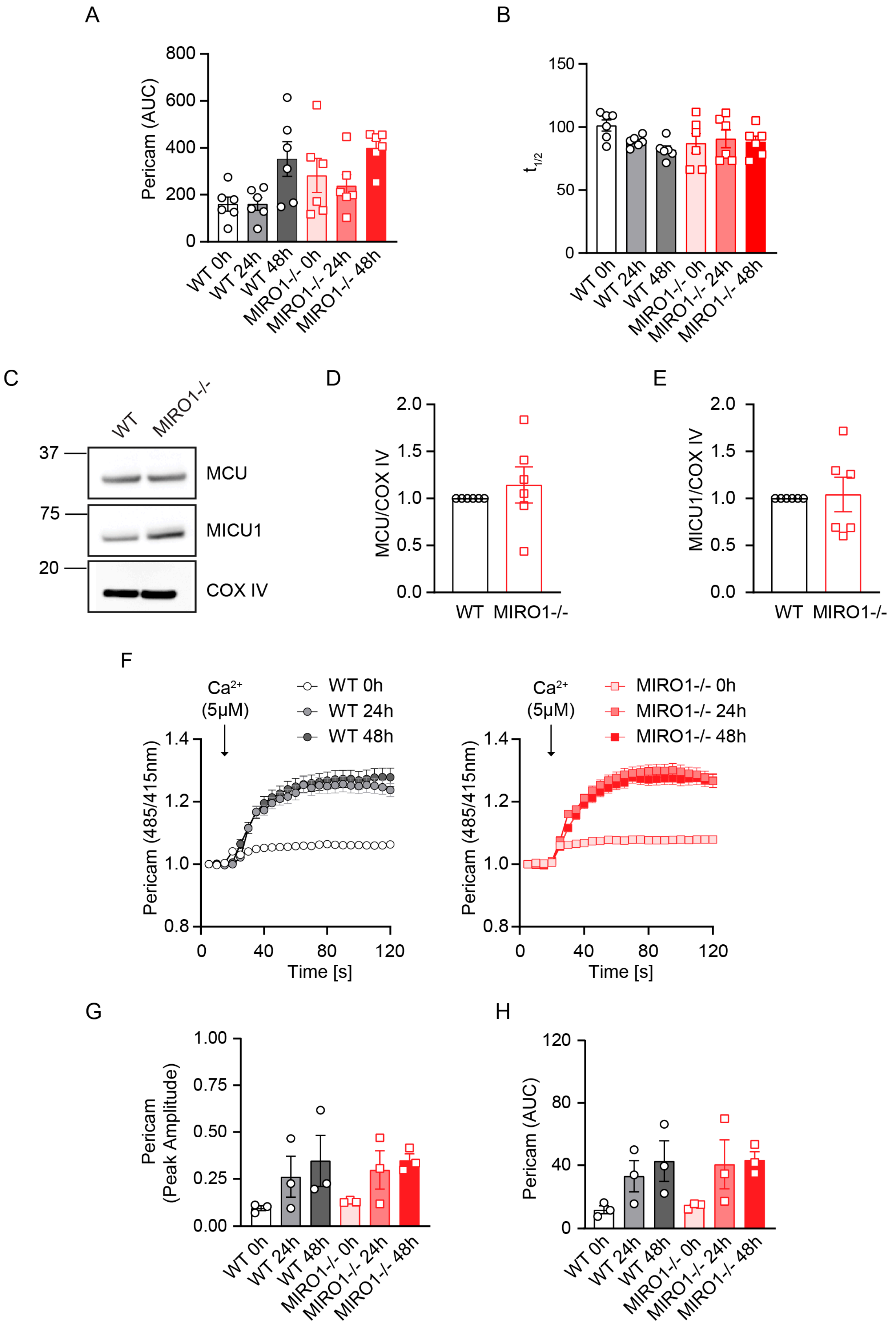
A
B
Pericam (AUC)
800
600
400
200
0
WT 0h
WT 24h
WT 48h
MIRO1-/- 0h
MIRO1-/- 24h
MIRO1-/- 48h
$t_{1/2}$
150
100
50
0
C
D
E
WT
MIRO1-/-
37
75
20
MCU
MICU1
COX IV
MCU/COX IV
MICU1/COX IV
2.0
1.5
1.0
0.5
0.0
F
Ca^{2+}
(5µM)
WT 0h
WT 24h
WT 48h
MIRO1-/- 0h
MIRO1-/- 24h
MIRO1-/- 48h
Pericam (485/415nm)
1.4
1.2
1.0
0.8
0
40
80
120
Time [s]
G
H
Pericam
(Peak Amplitude)
1.00
0.75
0.50
0.25
0.00
Pericam (AUC)
120
80
40
0

Figure S6: MIRO1 regulates changes in subcellular Ca^{2+} distribution during cell cycle but has no effect on MCU complex protein expression or direct mitochondrial Ca^{2+} entry in permeabilized VSMCs.

(A) Quantification of the area under the curve (AUC) for mitochondrial Ca^{2+} transients shown in Figure 4E; n = 6 independent experiments.

(B) Quantification of time-to-peak for mitochondrial Ca^{2+} transients shown in Figure 4E; n = 6 independent experiments.

(C) Representative immunoblots for MCU complex proteins in mitochondrial fractions from WT and MIRO1$^{-/-}$ VSMCs; COX IV was used as loading control.

(D, E) Quantification of immunoblot experiments shown in (C). (D) MCU, (E) MICU1, adjusted to COX IV; n = 6 independent experiments.

(F) Mitochondrial Ca^{2+} uptake assessed with mtPericam in response to $CaCl_2$ (5 μM) in synchronized/ growth arrested WT and MIRO1$^{-/-}$ VSMCs at 0 h, and after release from arrest with 10% FBS for 24 h, and 48 h. Cells were permeabilized before recordings. Arrows indicate addition of Ca^{2+}.

(G) Quantification of peak amplitude of mtPericam recordings shown in (F); n = 3 independent experiments.

(H) Quantification of the area under the curve (AUC) shown in (F); n = 3 independent experiments.

Data are shown as the mean ± SEM. Analyses were performed using the Kruskal-Wallis (A - B, G - H), and Wilcoxon (D, E) tests.

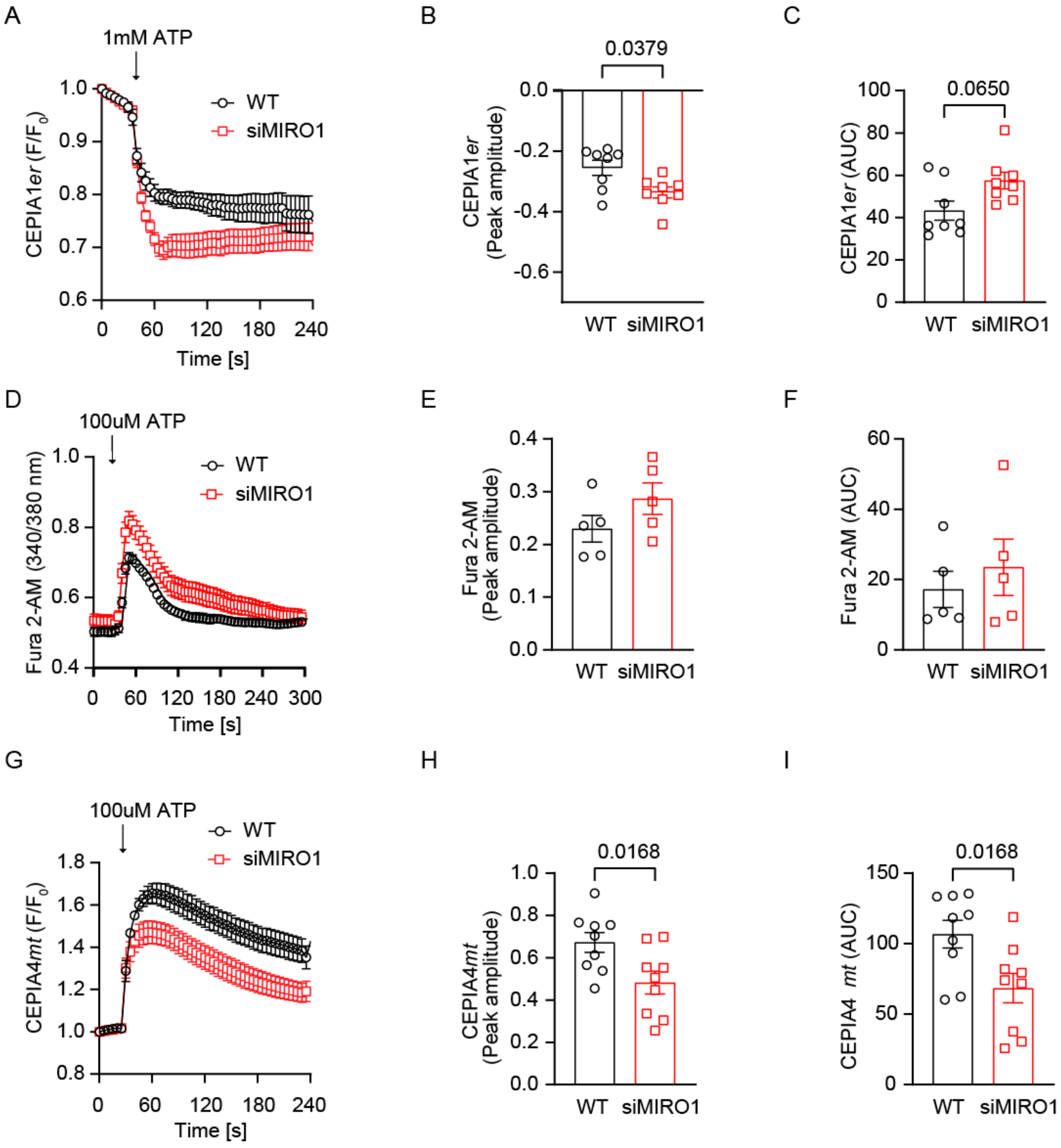

Figure S7: Acute knockdown of MIRO1 with siRNA impaired ER-mitochondria Ca^{2+} transfer in HEK cells.

(D) Agonist-induced ER Ca^{2+} release as assessed with CEPIA1*er* in WT and siMIRO1 cells. Arrow indicates addition of agonist, ATP (1 mM).

(B, C) Quantification of the peak amplitude and area under the curve (AUC) of CEPIA1*er* recordings shown in (A); n = 8 independent experiments.

(D) Agonist-induced cytosolic Ca^{2+} transients as assessed with Fura 2-AM in WT and siMIRO1 cells. Arrow indicates addition of agonist, ATP (100 µM).

(E, F) Quantification of the peak amplitude and area under the curve (AUC) of Fura 2-AM recordings shown in (D); n = 5 independent experiments.

(G) Agonist induced mitochondrial Ca^{2+} uptake as assessed with CEPIA4*mt* in WT and siMIRO1 cells. Arrow indicates addition of agonist, ATP (100 µM).

(H, I) Quantification of the peak amplitude and area under the curve (AUC) of CEPIA4*mt* recordings shown in (G); n = 8 independent experiments.

Data are shown as the mean ± SEM. Analyses were performed using the Mann-Whitney (B, C, E, F) and unpaired t (H, I) tests.

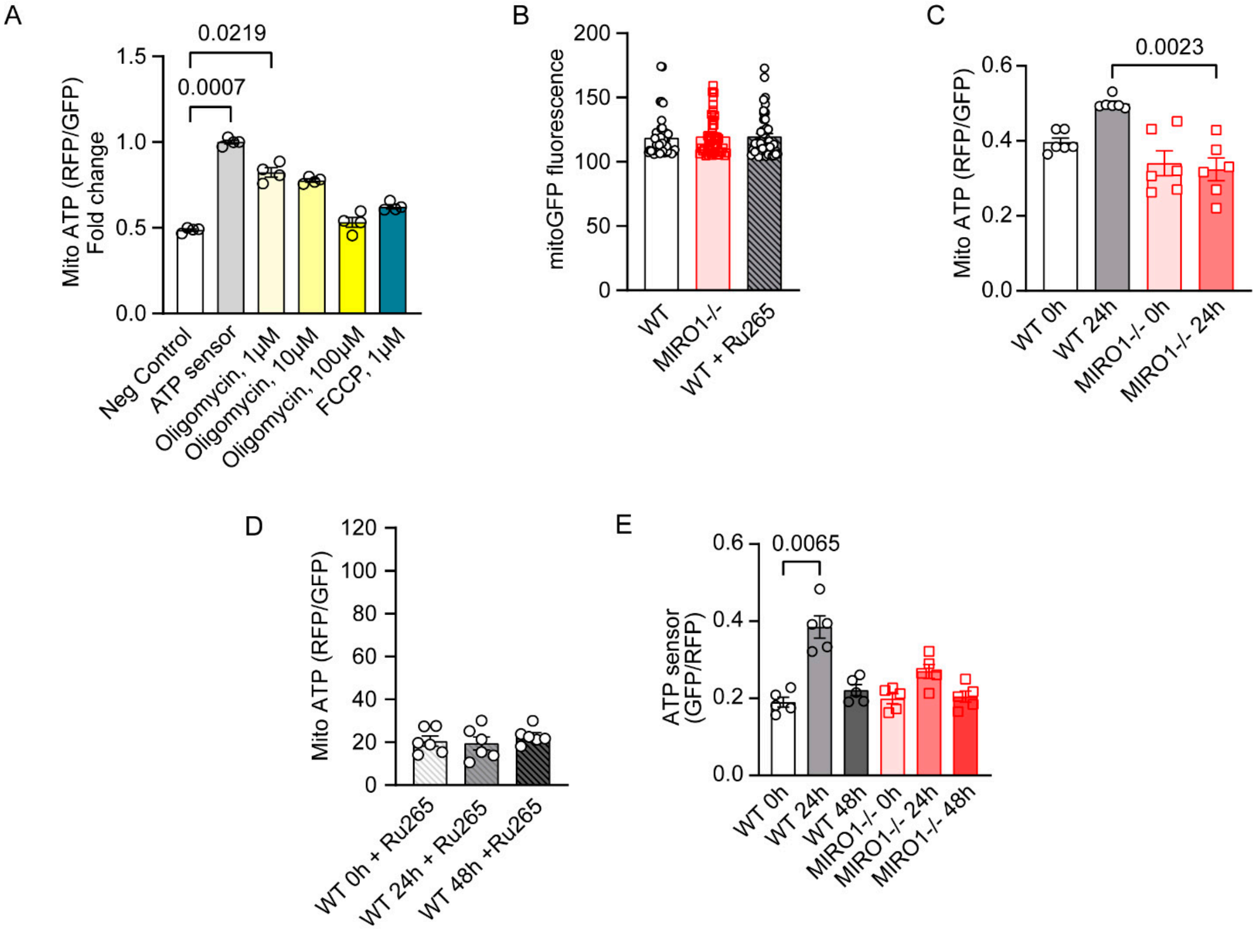

Figure S8: ATP-Red live cell dye selectively detects mitochondrial ATP.

A) Quantification of mitochondrial ATP in VSMCs treated with oligomycin (1 µM, 10 µM, and 100 µM) overnight or FCCP (10 µM) for 30 min; n = 4 technical replicates.

B) Quantification of mitoGFP fluorescence in WT and MIRO1$^{-/-}$ VSMCs and WT VSMCs treated overnight with Ru265 (100 µM) and incubated with ATP-Red live cell dye; n = 50 cells per group.

C) Quantification of mitochondrial ATP levels in synchronized/growth arrested WT and MIRO1$^{-/-}$ skin fibroblasts at 0 h, and after release from arrest with 10% FBS for 24 h; n = 6 independent experiments.

D) Quantification of mitochondrial ATP levels in synchronized/growth arrested WT VSMCs at 0 h, and after release from arrest with 10% FBS for 24, and 48 h treated overnight with Ru265 (100 µM); n = 6 independent experiments.

E) Quantification of cytosolic ATP levels in synchronized/growth arrested WT and MIRO1$^{-/-}$ skin fibroblasts at 0 h, and after release from arrest with 10% FBS for 24 h, and 48 h; n = 5 independent experiments.

Data are shown as the mean ± SEM. Analyzed using Kruskal-Wallis test.

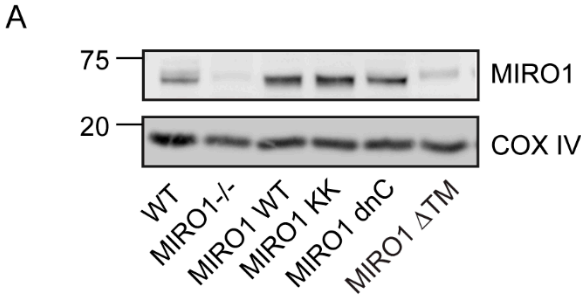

Figure S9: Expression of MIRO1 WT, MIRO1 KK, and MIRO1 dnC in MIRO1$^{-/-}$ skin fibroblasts.

(I) Representative immunoblot of MIRO1 in mitochondrial fractions of WT skin fibroblasts and MIRO1$^{-/-}$ skin fibroblasts transduced with adenovirus expressing MIRO1 WT, MIRO1 KK, MIRO1 dnC, or MIRO1 ΔTM for 72 h; COX IV was used as loading control.
